# Development and Validation of Chinese University Students’ Physical Activity Motivation Scale Under the Constraint of Physical Education Policies

**DOI:** 10.3389/fpsyg.2022.722635

**Published:** 2022-02-25

**Authors:** Bo Lin, Eng Wah Teo, Tingting Yan

**Affiliations:** ^1^Centre for Sport and Exercise Sciences, University of Malaya, Kuala Lumpur, Malaysia; ^2^School of Physical Education, Henan Institute of Science and Technology, Xinxiang, China

**Keywords:** physical activity, motivation, physical education policy, validity, reliability

## Abstract

The accurate measurement of university students’ motivation to participate in physical activity (PA) is a prerequisite to developing better physical fitness programs. However, motivation driven by government policies, i.e., physical education policies, are often excluded from many existing scales. The purpose of this study was to develop and evaluate a psychometric instrument based on self-determination theory that exclusively measures the motivation of Chinese university students to participate in PA. A total of 1,215 university students who regularly participated in PA at five universities in China constituted the final valid sample. Sample 1 (*n* = 311) was used to determine the underlying factor structure of the initial Chinese University Students’ Physical Activity Motivation Scale (CUSPAMS) through exploratory factor analysis (EFA). Sample 2 (*n* = 330) was used to test the model fit of the EFA-derived factor structure and data through confirmatory factor analysis (CFA) and to test the internal consistency of each factor and of the whole scale. Sample 3 (*n* = 574) was used to confirm the model stability and criterion validity. Finally, 177 individuals were randomly selected from Sample 3 to perform test–retest reliability. Preliminary evidence showed that the nine-factor CUSPAMS, consisting of 32 items, yielded good psychometric characteristics. The development of the CUSPAMS provides an opportunity to improve current theories and practices regarding the assessment of PA motivation. The CUSPAMS is recommended for examining factors that influence motives as well as the impact of motives on PA among Chinese university students.

## Introduction

Although the benefits of physical activity (PA) and exercise have been demonstrated across the lifespan, physical inactivity is still a major global concern, one that threatens health worldwide (Kohl et al., 2012). In many countries, more than 50% of university students fail to achieve at least 150 min of moderate-intensity PA per week (Hoyos et al., 2011; Small et al., 2013). Similarly, this trend also occurs in China—for example, in 2019, out of a total of 30.31 million Chinese college and university students (Ministry of Education of the People’s Republic of China, 2020), approximately 75% failed to accomplish a minimum of 150 min of moderate-intensity aerobic activity per week or 75 min of vigorous aerobic activity per week (Wang, 2016). According to the “2014 National Physical Fitness and Health Surveillance” report, the prevalence of PA time less than 1 h per day was high in students aged 9–22 years. Among them, the 18-year-old male group and the 21-year-old female group had the highest prevalence, 82.5 and 89.8%, respectively (Wang et al., 2017). Wu et al. (2015) found that an increase in sedentary lifestyles and reduced PA are becoming major health concerns for Chinese university students. These unhealthy lifestyles, formed during university careers, usually persist into later life and lead to long-term negative health consequences (Friedman et al., 2008). According to data from the seven National Student Physical Fitness and Health Surveys from 1985 to 2014, the physical fitness of university students is exhibiting a downward trend, and the obesity rate continues to increase by 2–3% every 5 years (Chinese Students Physical and Health Research Group, 2018).

Motivation has been widely acknowledged as one of the key elements leading to persistent physical activities and exercise (Wilson and Rodgers, 2007). However, empirical research on the motivation of university students is limited. Despite the abundance of recent motivation studies, no scales have been specifically designed and validated to assess PA motivation, especially in the Chinese language. In a few recent studies, however, psychologists have recognized that cultural differences can influence the motivational climate of PA, which can also affect one’s perception of motivation (Gurleyik, 2012). For example, the association between PA and health motivation varies significantly by region (i.e., North America, Eastern Europe, and Western Europe) and gender (Iannotti et al., 2012). Therefore, the social and culture environments of sample populations are factors that must be given ample consideration in any scale development.

In the current study, policy-driven PA was motivated by the physical education (PE) sector of school management to cater to the needs of the Chinese population. Since the 1950s, the Chinese government has implemented various PE system reformations through the promulgation of PE policies designed to address challenges related to physical inactivity in schools. The formulation of these PE policies is aimed at promoting and improving the physical health of Chinese students. From elementary school to university, every component of PE, such as the PE curriculum and extracurricular sports, are implemented by schools in accordance with the policy documents issued by the government. Table 1 lists the main policy documents and contents issued by the Chinese government from 2002 to 2019 to improve university students’ level of PA. In recent years, as local colleges and universities have implemented increasingly stringent national PE policies, students have passively participated in PA and exercise in order to pass the national physical fitness test standards required in the national PE policy documents (Chen et al., 2008; CGTN, 2018). Obviously, the educational background that gestates policy motives has been excluded from previous motivation scales. Therefore, motivation scales derived from existing educational systems have tended to have certain limitations when applied in China.

**Table 1:** Physical education (PE) policies issued by the Chinese government since 2002 for university students.

Policy	Year	Main content
Physical Education Curriculum Teaching Guidelines for Common Institutes of Higher Learning in China	2002	(1) First and second grades of common universities must offer PE courses (four semesters, total of 144 credits). (2) Universities should offer optional PE courses for students above second grade (including graduate students). Completing the required credits and meeting the basic requirements are necessary to graduate and attain a degree.
National Student Physical Health Standard	2002	(1) From 2004, university students must participate in physical fitness tests organized by their universities every academic year. (2) Test contents are introduced, including the three required test items and three selected test items. (3) Students with “good” or “excellent” physical fitness are eligible for scholarships or other awards, with 60 points on physical health tests needed to graduate.
Revised National Student Physical Health Standard (2007 version)	2007	(1) Adjusted selected test items and corresponding test scores. (2) Adjusted physical fitness test scores.
Hundreds of Millions of Students Nationwide Sunshine Sports	2007	Requires 85% of students to exercise one hour per day, master two sports skills, and form a habit of physical exercise within 3–5 years.
Basic Standards for Physical Education in Colleges and Universities	2014	(1) Universities must ensure that PE courses for students include no fewer than two credits per week, with each credit no fewer than 45 min. (2) University students participate in at least three extracurricular workouts per week. (3) Universities should ensure that students have one hour of physical activity (PA) every day.
Revised National Student Physical Health Standard (2014 version)	2014	(1) The university student physical fitness test was changed to include seven mandatory test items. (2) Under the new standard, a test score of good or above is needed to participate in honorary evaluations. (3) University students with a total score of fewer than 50 points on the sports test will not get a diploma.
Opinions of the Ministry of Education on Deepening the Reform of Undergraduate Education and Teaching to Improve the Quality of Talent Cultivation	2019	The 2019 Reform stated that university students who fail to meet the “National Student Health Standard” cannot graduate.

The above documents are issued by the Ministry of Education of the People’s Republic of China.

Over the past 50 years, several motivation scales have been developed by various researchers across the globe to measure motivations for exercise and sports engagement (Bartholomew et al., 2009; Table 2). In general, two main approaches are favored by sports psychologists in developing instruments, i.e., a theoretical approach (based on existing theories) and an atheoretical approach (interviews with a target population; Molanorouzi et al., 2014). However, the scales developed based on these two approaches are too specific, consequently limiting general or practical applications (Vallerand and Fortier, 1998; Clancy et al., 2017). For example, the Sport Motivation Scale (SMS; Pelletier et al., 1995), the Behavioral Regulation in Sport Questionnaire (BRSQ; Lonsdale et al., 2014), and the Perceptions of Success Questionnaire (POSQ; Roberts et al., 1998) were designed specifically for use in the competitive sports context. The Exercise Motivation Scale (EMS; Li, 1999) was designed to evaluate the simplex mode of the self-determination continuum under various motivational orientations. The Situational Motivation Scale (SIMS; Guay et al., 2000), however, is not specifically a sports questionnaire and can be applied across diverse domains. The limitation of the SIMS is that intrinsic motivation is assessed unidimensionally, while two types of extrinsic regulations are not taken into consideration (Clancy et al., 2017). The Intrinsic Motivation Inventory (IMI; McAuley et al., 1989) is mainly used to assess the determinants and consequences of intrinsic motivation, rather than intrinsic motivation itself, and there is no factor for extrinsic motivation. An example of scale development that uses an atheoretical approach is the Participation Motivation Questionnaire (PMQ; Gill et al., 1983). The PMQ aims to examine the motives for participation in different contexts in the fields of exercise and sports, but Frederick and Morris (2004) deemed that a stable version of the PMQ containing a set number of items that can be used in a variety of PA contexts has not been established to date.

**Table 2:** Overview of motivation measures in sports and PA.

Scale	Factors	Items	Likert scale
Sport Motivation Scale (SMS; Pelletier et al., 1995)	7	28	1–7
Sport Motivation Scale-6 (SMS-6; Mallett et al., 2007)	6	24	1–7
Revised Sport Motivation Scale (SMS-II; Pelletier et al., 2013)	6	18	1–7
Exercise Motivation Inventory (EMI; Markland and Hardy, 1993)	12	44	1–6
Exercise Motivation Inventory-2 (EMI-2; Markland and Ingledew, 1997)	14	69	1–6
Motivation for Physical Activity Measure (MPAM; Frederick and Ryan, 1993)	3	23	1–5
Motivation for Physical Activity Measure—Revised (MPAM-R; Richard et al., 1997)	5	30	1–7
Intrinsic Motivation Inventory (IMI; McAuley et al., 1989)	4	16	1–7
Exercise Motivation Scale (EMS; Li, 1999)	8	31	1–6
Situational Motivation Scale (SIMS; Guay et al., 2000)	4	14	1–7
Perceptions of Success Questionnaire (POSQ; Roberts et al., 1998)	2	12	1–5
Task and Ego Orientation in Sport Questionnaire (TEOSQ; Duda, 1989)	2	13	1–5
Behavioral Regulation in Sport Questionnaire (BRSQ; Lonsdale et al., 2014)	6	24	1–7
Participation Motivation Questionnaire (PMQ; Gill et al., 1983)	8	30	1–3
Recreational Exercise Motivation Measure (REMM; Rogers, 2000)	8	73	1–5
Physical Activity and Leisure Motivation Scale (PALMS; Morris and Rogers, 2004)	8	40	1–5

The authors reviewed 16 articles on PA motivation published in core sports journals in China over the past decade and found that 15 of these articles employed scales developed based on non-local samples. Among them, the Chinese version of the MPAM-R and the PALMS are the most commonly used instruments to measure PA motivation among the Chinese population (Zhu et al., 2016; Zhu and Dong, 2016). However, aside from the fact that these two scales do not include policy-related motivations, they have other limitations. For example, the MPAM-R contains only five types of motivation: fitness, appearance, competition, social, and enjoyment. This prevents some real motivations from being detected by this scale. As for the PALMS, the target population is recreational exercise participants (Roychowdhury, 2012) or individuals who work in various organizations (Zach et al., 2012). This consequently leads to a description of some items on the scale that does not match the actual age group of university students.

Therefore, the paucity of validated measures of PA motivation developed for university students appears to represent a critical barrier to accurately understanding their motives for engaging in PA and to developing strategies for effectively promoting PA for this particular population. Considering the aforementioned issue, the purpose of this study was to develop a Chinese localized questionnaire specifically targeted to measure the motivation for PA participation among university students under the influence of China’s PE policies. The information provided by this study and the use of this new scale will help researchers better understand the current status of university students’ motivation to participate in PA. Second, this work will reveal how these policies affect university students’ PA participation and may improve PA participation among university and college students.

### Framework and Hypotheses

The quality and type of exercise motivation play a vital role in the success or failure of healthy behavioral change. Self-determination theory (SDT) provides a framework for explaining human behavior and motivation in sports and exercise (Deci and Ryan, 1985, 2000). According to SDT, there are three main types of human motivation in the field of exercise—namely, amotivation, extrinsic motivation, and intrinsic motivation. These three motivations are distributed on the motivation continuum according to the degree of self-determination. Amotivation and intrinsic motivation are located at the two extremes of the continuum, representing the most controlled and autonomous forms of motivation, respectively. Extrinsic motivation, situated between amotivation and intrinsic motivation on the continuum, refers to performing activities for instrumental reasons or achieving outcomes separate from the behavior itself. In the sub-theory of SDT, organic integration theory (Ryan and Deci, 2000), external motivation is further conceptualized into four types—namely, external regulation, introjected regulation, identified regulation, and integrated regulation. In the physical exercise domain, these types differ in their relative autonomy. External regulation occurs when individuals engage in exercise to fulfill external demands, obtain rewards, or avoid punishment, such as exercising in order to please others. Introjected regulation occurs when exercise is performed to avoid feelings such as guilt or shame or to enhance ego and feelings of self-worth, such as exercising in order to lose weight or improve body shape. These two external motives represent those described as controlled forms in SDT, which are not or are only partially internalized. Although they sometimes regulate (or motivate) short-term exercise behavior, they do not sustain maintenance over time (Deci and Ryan, 1985). In contrast, identified regulation and integrated regulation represent a more autonomous form of exercise regulation. Identified regulation exists when an individual participates in exercise not because of the fun and satisfaction of the behavior itself but instead because of its recognized health value and utility (Ryan et al., 2009). Integrated regulation is a type of regulation in which an individual regards exercise not only as individually essential but also as congruent with deeply held values and sense of self. However, exercise undertaken to achieve an external goal—creating or confirming one’s identity—is still considered external regulation.

Deci and Ryan (2008) suggested that more self-determined regulation is associated with greater persistence, enhanced performance, and better well-being. Similar to behavioral regulation, different types of motives may differ in their degree of internal or external orientation (Markland and Ingledew, 2007). Within the exercise domain, external goals (e.g., weight loss, appearance) are always considered to be associated with less self-determined regulation, while internal goals (e.g., health, affiliation) are always associated with more self-determined regulation (Gillison et al., 2006; Ingledew and Markland, 2008). In the MPAM-R, exercise for health and fitness is classified as controlled-oriented motives, and exercise for social interaction and competition is classified as autonomous motivation. Only the latter can significantly predict subsequent PA. However, unlike behavioral regulation, PA motives deal with the goal content—that is, they focus on what exercise-related goals people want to pursue (Deci and Ryan, 2000). On the contrary, behavioral regulation is more concerned with the why of goal pursuit—that is, the autonomous and controlled motivations that guide people’s efforts to achieve a certain goal.

**Figure 1:**
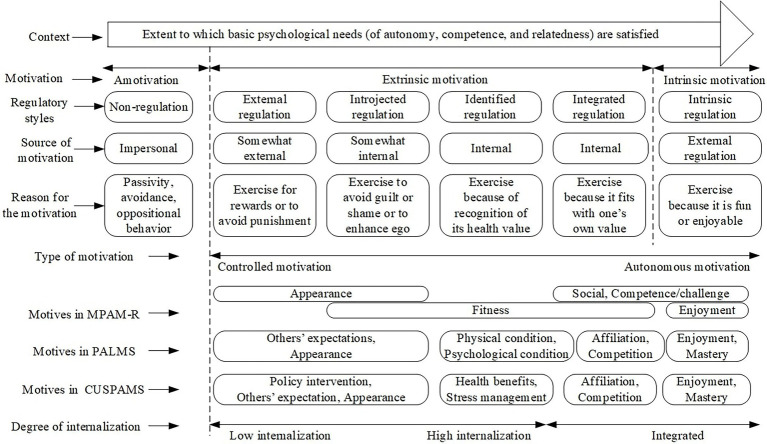
Theoretical framework of Chinese University Students’ Physical Activity Motivation Scale (CUSPAMS). Source: adapted from self-determination theory (Ryan and Deci, 2000).

This research project sought to develop and validate a Chinese University Students’ Physical Activity Motivation Scale (CUSPAMS) to measure various forms of motivational content geared toward performing healthy behaviors in the SDT paradigm. Figure 1 uses the form of a continuum to show the antecedents, perceived autonomy, and internalization of these different content motivations. In addition, it also describes the differences and connections between the MPAM-R, PALMS, and CUSPAMS in measuring motivational content and highlights the extent of relative autonomy of these motivations. The following hypotheses were put forward: In terms of structural validity, we assumed that the CUSPAMS would have nine factors and that the motivation goals mentioned in SDT would receive responses by the sample of Chinese university students (H1). As for criterion validity, two hypotheses were formulated: We expected that the CUSPAMS would be correlated with the selected scales: the revised version of the Behavioral Regulation in Exercise Questionnaire (BREQ-2; Markland and Tobin, 2004) and the Self-Efficacy for Exercise (SEE) Scale (Resnick and Jenkins, 2000). Specifically, we expected that the factors in the CUSPAMS, such as enjoyment, mastery, affiliation, and competition, would be highly positively correlated with the autonomous form of motivational regulation (e.g., identified and intrinsic motivation), while appearance, policy-related motives, and others’ expectations would be highly positively correlated with controlled forms of motivational regulation (e.g., amotivation, external and introjected regulation; H2). Based on the literature report (McAuley et al., 1991c^1994^; Kavussanu and Roberts, 1996; Slovinec D’Angelo et al., 2014), we also expected that in the CUSPAMS, more autonomous motivation factors would have a higher positive correlation with exercise self-efficacy, while controlled motivation factors would have a lower positive correlation or no correlation (H3).

## Materials and Methods

### Development of the CUSPAMS

The CUSPAMS was designed to assess types of motivation among university students engaged in PA. In the current study, the scale development process included the following procedures. First, a comprehensive iterative review and in-depth content analysis were conducted in order to identify and describe the different aspects of university students’ motivation to participate in PA. Depending on common agreement among studies on PA motivation and drawing on studies of the motivation of Chinese scholars, nine dimensions were proposed: (1) stress management, (2) competition, (3) appearance, (4) affiliation, (5) enjoyment, (6) others’ expectations, (7) mastery, (8) health benefits, and (9) policy intervention.

Second, once the dimensions were identified, item pools were generated using the “deductive” method recommended by Raykov and Marcoulides (2011)—that is, through a literature review and assessment of existing scales and indicators in the field of sports and exercise. The authors of this study invited five experts from the fields of PE, sport psychology, linguistics, and Chinese school PE policy to conduct three rounds of online meetings to discuss and determine the items in the new scale. In the first meeting round, the expert panel reviewed the previous 16 motivational scales and concluded that the items in the MPAM-R and PALMS were a suitable basis for item development. There were three reasons for this conclusion: First, the two scales are derived from classic SDT as the theoretical basis for developing the structure of the scale; second, the items in these two scales describe specific motivational goals in the PA domain; third, these two scales have demonstrated good psychometric characteristics in different cultures. In the second meeting round, the panel compared the verbal expression of both scales to extract a series of items that reflected the same factors but used different terminologies in these two scales. For example, the statement “Because I want to be physically fit” in the MPAM-R is similar to “be physically fit” in the PALMS, as both reflect the dimension of health motivation—therefore, this item was retained. Items that did not reflect the actual context of Chinese university students’ PA participation, such as “to earn a living” and “because I get paid to do it” in the PALMS, were deleted after discussion between the experts. Besides, with reference to the views put forward by Chinese scholars (Chen et al., 2008; He and Yang, 2018), the authors drafted four policy intervention motivation items. Then, the expert panels, especially policy experts and linguists, discussed and reviewed the content and sentences of the four items. In the third meeting round, the panel members conducted a comprehensive evaluation of the conceptual scope, semantic equivalence, clarity, readability, relevance, and conciseness of the initial items. Three items with low clarity were deleted in this round. After all panel members agreed that none of the remaining items required further modification, 41 items were generated from three extraction methods (joint extraction of two scales, item extraction from a single scale, and self-developed items from the Chinese literature; Table 3), together comprising the first version of the CUSPAMS.

**Table 3:** Initial 41 items of the CUSPAMS.

Initial item pool	Factor
01. Because I want to be physically fit.^a^	Health benefits
10. Because I want to have more energy.^a^	
19. Because I want to improve my cardiovascular fitness.^a^^,^^b^	
20. I want to be fitter than others.^a^	
28. Because it helps to maintain a healthy body.^a^	
36. Because I want to prevent disease through participating in physical activities.^c^	
02. Because I like to engage in activities that physically challenge me.^b^	Mastery
07. Because I want to improve my existing skills.^a^^,^^b^	
11. Because I want to get better at my activity.^a^^,^^b^	
16. Because I want to keep up my current skill level.^b^	
25. Because I want to obtain new skills.^a^^,^^b^	
33. Because I want to test the limits of my abilities.^c^	
29. Because I want to compete with other people around me.^a^	Competition
37. Because I want to show my athletic ability to others.^c^	
39. Because participating in physical activities helps me get more opportunities (e.g., promotion to graduate student) and honors (e.g., scholarships).^c^	
41. Because people around me reward me when I do.^c^	
04. Because it is more fun to exercise with others.^c^	Affiliation
13. Because I like to be with others who are interested in this activity.^b^	
22. Because I want to meet new people.^b^	
31. Because I want to do something in common with friends.^a^	
05. Because it makes me happy.^a^^,^^b^	Enjoyment
14. Because it is fun.^a^^,^^b^	
23. Because I think it is interesting.^a^^,^^b^	
32. Because I enjoy this activity.^a^^,^^b^	
38. Because I like the excitement of participation.^b^	
08. Because it helps me relax.^a^	Stress management
17. Because it acts as a stress releaser.^a^	
26. Because it helps me to get away from pressures.^a^	
34. Because it helps me take my mind off other things.^a^	
06. Because my friends want me to.^b^	Others’ expectations
15. Because people tell me I need to.^a^	
24. Because it was prescribed by a doctor or physiotherapist.^a^	
09. Because I want to meet the physical activity standard required by the university.^c^	Policy intervention
18. Because I want to pass the minimum score required by the national physical fitness test.^c^	
27. Because I want to get credits for physical education class.^c^	
35. Because I want to get a high score on the national physical fitness test.^c^	
03. Because I want to lose weight so that I look better.^a^^,^^b^	Appearance
12. Because I want to define my muscles so that I look better.^a^^,^^b^	
21. Because I want to improve my body shape.^a^^,^^b^	
30. Because I want to maintain a trim, toned body.^a^	
40. Because I want to improve my appearance.^a^^,^^b^	

a Item taken from a Physical Activity and Leisure Motivation Scale (PALMS).

b Motivation for Physical Activity Measure—Revised (MPAM-R).

c Developed in the current study.

Finally, the face validity of the new scale items was obtained through extensive online comments by 20 Chinese university students (face validity). The purpose was to determine whether they understood the items and were able to respond to them. Their feedback indicated that no further modifications to the scale were needed.

### Participants

The initial version of the CUSPAMS was completed by 641 university students (194 males and 447 females), with a mean age of 20.23 ± 1.46 years, from five universities in five different provinces located in central China. Of these students, 301 were freshmen (46.9%), 176 were sophomores (27.4%), 56 were juniors (8.7%), and 88 were seniors (13.7%). Various majors were represented by these students. The most common physical activities reported were walking (*n* = 200), jogging (*n* = 175), and basketball (*n* = 32). The participants reported an average of 1.68 ± 1.66 h of PA per week. For subsequent data analysis, the participants were randomly split into two subsamples: Sample 1 (*n* = 311; male = 89, female = 222; *M* = 20.17 ± 1.39) was used to develop the initial scale and to examine its underlying factor structure by exploratory factor analysis (EFA); Sample 2 (*n* = 330; male = 105, female = 225; *M* = 20.28 ± 1.53) was used to test for data fit by confirmatory factor analysis (CFA). To test the model stability and criterion validity, 574 university students (Sample 3; Mage = 19.21 ± 1.23, male = 314) from a public university in Henan Province voluntarily completed a survey, including demographic information, and three scales at the same time. To measure the test–retest reliability of the CUSPAMS, each participant in Sample 3 was assigned a unique questionnaire code, and each was advised to refill in the same questionnaire using their unique code 21 days later, i.e., to complete the survey a second time. Finally, 177 participants were randomly selected from Sample 3 for test–retest process.

In this study, we established the following inclusion criteria for the participants: (1) had to be a registered university student, (2) had to be at least 18 years old, (3) had to be non-sport/PE major students, (4) had to be Chinese, (5) had to have a strong comprehension of the Chinese language in terms of reading, speaking, and writing, and (6) had to regularly participate in physical activities at least once per week for the past 3 months. And, finally, since the questionnaire was conducted online, the participants (7) had to be smartphone users. The exclusion criteria were as follows: (1) cannot participate in regular exercise due to physical disability, or (2) unwilling to participate in the study.

### Procedure

Hall et al. (2020) mentioned that the ongoing impact of the COVID-19 pandemic is changing people’s exercise behavior. A long-term home isolation lifestyle has led to more physical inactivity and increased sedentary behavior. To reduce these impacts as much as possible, the online questionnaire was distributed in August 2020, at which time it had been at least 3 months since the participants’ lives had returned to normal in China. University students had also returned to their respective universities. The questionnaire was distributed through “Questionnaire Star,” a mobile application specializing in online surveys that is widely used in China. Prior to online survey administration, ethical approval was obtained from the Ethics Committee of the University of Malaya (UM. TNC2/UMREC-976). Since the actual data collection was in China, research permission was also obtained from the Student Affairs Office of the five targeted Chinese universities. The researchers then contacted administrators and PE lecturers and explained the objectives and other vital information related to the study. With their approval, they shared a QR code that included all online questionnaires to WeChat (a popular social chat application in China, similar to WhatsApp) groups of students from different study years and majors. These WeChat groups were created by administrators and PE lecturers after the students had enrolled in the university with the purpose of sharing physical class or academic-related information. Online consent to participate was obtained when the participants volunteered to scan the QR code and complete the online questionnaire. The participants were fully informed that they could withdraw from the study at any time during the process of completing the online questionnaires and that all of the information they provided would remain confidential. The online questionnaire took approximately 3–6 min to complete. After the participants had completed the CUSPAMS online questionnaire, their responses were automatically saved in the “Questionnaire Star” application, after which the researchers downloaded the collected data directly from the application.

Data collection for Sample 3 occurred in November 2021 using a method similar to that for Sample 1 and Sample 2. In addition to completing the CUSPAMS, Sample 3 also participated in two other instruments to test the criterion validity of the CUSPAMS, including the BREQ-2 developed based on SDT and the SEE Scale. In previous studies, behavioral regulation and self-efficacy were demonstrated to have a strong correlation with motivation types (Mullan and Markland, 1997; Ryan and Deci, 2000; Teixeira et al., 2012).

### Measures

The participants reported key demographic information, including gender, age, university affiliation, major/field of study, and number of years in university. They also reported the frequency of their regular weekly PA, its duration, and its intensity during the past 3 months.

The newly developed 41-item CUSPAMS was used to identify the perceived reasons for participating in PA. The participants were asked to respond to questions such as the following: “Why do you participate in physical activity?” The online scale included motives for nine factors. For example, “Because I want to be physically fit” is an example of an item from the health benefits factor (six items); “Because I like to engage in activities that physically challenge me” is an example of an item from the mastery factor (six items); “Because I want to compete with other people around me” is an example of an item from the competition factor (four items). All items were based on a 7-point Likert scale ranging from 1 (not at all true for me) to 7 (very true for me), indicating the degree to which each motive was personally true for each participant with respect to primary PA.

The BREQ-2 (Markland and Tobin, 2004) was employed to measure behavioral regulation in exercise and has been found to be reliable and valid among Chinese university students (Liu et al., 2015). The C-BREQ-2 consists of 18 items with responses on a 5-point Likert scale ranging from 0 (not true for me) to 4 (very true for me). Five subscales are included in this scale: amotivation, with four items (e.g., “I do not see why I should have to exercise”), external regulation, with four items (e.g., “I exercise because others will not be pleased with me if I do not”), introjected regulation, with three items (e.g., “I feel like a failure when I have not exercised in a while”), identified regulation, with three items (e.g., “It’s important to me to exercise regularly”), and intrinsic regulation, with four items (e.g., “I get pleasure and satisfaction from participating in exercise”). A higher score on the identified regulation and intrinsic motivation subscale indicated that the exerciser’s motivational form tended to be more autonomous. The Cronbach’s *α* for the C-BREQ-2 was 0.90.

The SEE Scale was designed to evaluate people’s confidence to continue exercising in the face of barriers to exercise (Resnick and Jenkins, 2000). The reliability and validity of the Chinese version of the SEE (SEE-C) was provided by Lee et al. (2009). The SEE-C comprises nine items with ratings on a 10-point Likert scale ranging from 0 (not confident) to 10 (very confident). Higher scores indicate higher levels of self-efficacy. The Cronbach’s *α* for the SEE-C was 0.91.

### Statistical Analysis

Exploratory factor analysis and CFA are widely used in measurement applications for scale development and construct validation (Ye et al., 2018a; Chang et al., 2020; Fung and Fung, 2020). The data from the initial samples were randomly divided into two groups using SPSS, i.e., Sample 1 for EFA and Sample 2 for CFA. Descriptive statistics of the items of the CUSPAMS and the bivariate correlations between items were computed using data from Sample 1 (*n* = 311). Kolmogorov–Smirnov, skewness, and kurtosis tests were employed to check for data normality. The data were shown to be normally distributed. Before further analysis, Kaiser-Meyer-Olkin (KMO) and Bartlett’s sphericity tests were run to measure for sampling adequacy. Subsequently, the factorial structure of the CUSPAMS was tested using EFA, and internal consistency was estimated. There is currently no consensus on the sample size standard for variants of factor analysis but, according to suggestions by Pearson and Mundform (2010), a sample of 200 people was determined to be a sufficient minimum. The communality for each item was set to be greater than or equal to 0.40 (Leimeister, 2010) to confirm that each item shared some common variance with other items. Theoretical understanding and parsimony are considered when an item is loaded onto different factors (Kline, 2015). The reliability of the CUSPAMS was obtained by measuring the Cronbach’s *α* and the McDonald’s omega (*ω*; McDonald, 1999). This is because the latter can overcome some of the shortcomings of using *α* and thus represents one of the best measures of reliability (Yang and Green, 2011; Goodboy and Martin, 2020). Reliability measures less than 0.70 are considered moderate, 0.70–0.80 are considered sufficient, and more than 0.80 are considered good (Evers et al., 2010). In addition, we calculated the Pearson correlation coefficients between the CUSPAMS and the CUSPAMS completed after the 3-week interval to check test–retest reliability. The intraclass correlation coefficient value equal to or above 0.70 was considered acceptable (Weir, 2005).

Later, the structure of the CUSPAMS was further examined using CFA performed in SPSS AMOS version 22, based on data from Sample 2 (*n* = 330). CFA provides further evidence regarding the fitness of the suggested model with regard to the structure of the factors identified *via* EFA. The model parameters were estimated using the maximum likelihood function. Goodness of fit is a measure indicating how well a specified model reproduces the covariance matrix among the indicator variables (Hair et al., 2010). Multiple goodness of fit tests was used to evaluate the model’s fit to the data. To evaluate the fit of the models, we considered four indices of model fit: chi-square/degree of freedom (*χ*^2^/df), the comparative fit index (CFI), the Tucker-Lewis index (TLI), and the root mean square of error approximation (RMSEA). As the only true inference statistic that represents the model test (Markland, 2007), *χ*^2^ has been identified as potentially problematic due to sample size sensitivity, but its value was still reported. Marsh and Hocevar (1985) have recommended that a *χ*^2^/df value between 2.0 and 5.0 indicates a reasonable macro structure. As for the CFI and TLI, they should be ≥0.90 to indicate a good fit (Hair et al., 1998). The RMSEA value should be <0.05, as it can be said to indicate a convergent fit to the analyzed data of the model, while it also indicates a fit close to good when it produces a fit value between 0.05 and 0.08 (Browne and Cudeck, 1992).

## Results

### Exploratory Factor Analysis

Initially, the factorability of the 41 items was examined. Consistent with Field’s (2013) suggestion, the bivariate correlation matrix of all items was analyzed, and no items with a bivariate correlation score greater than 0.80 were found, thus avoiding the occurrence of multi-collinearity among items. The KMO value was 0.90, and Bartlett’s test of sphericity was significant (*χ*^2^ = 7,201.45, df = 820, *p* < 0.05), indicating that the samples met the criteria for factor analysis (Hair et al., 1998). However, item 24 (“Because it was prescribed by a doctor or physiotherapist”) and item 12 (“Because I want to define my muscles so that I look better”) showed communalities below 0.40. Osborne et al. (2008) stated that if communalities for a particular variable are low (between 0.00 and0.40), then that variable may struggle to load significantly on any factor. Thus, these two items were carefully observed in the subsequent EFA to determine whether to exclude or retain them. EFA was computed on the 41 items in the CUSPAMS for the 311 participants.

We follow the method provided by Tabachnick et al. (2007) to select the rotation of the factors. After requesting the oblique rotation, if the correlation between the factors exceeds 0.32, indicating that the variances between the factors partially overlap, it is more reasonable to use the oblique rotation, unless there is a convincing reason for the orthogonal rotation. Given that the correlation between the eight factors is between 0.20 and 0.40, a principal axis analysis with Promax rotation was performed. In addition, items that were found to cross-load or factor loadings lower than 0.40 were removed (Field, 2009), as were items that loaded ambiguously (i.e., the literal meaning of an item was clearly different from those of other items in the same factor). The remaining items underwent subsequent analyses, and inspections of the loadings were conducted. This iteration process was repeated until each item loaded significantly on only one factor with no cross-loadings. For example, item 24 (“Because it was prescribed by a doctor or physiotherapist”), item 22 (“Because I want to meet new people”), and item 14 (“Because it is fun”) were deleted due to factor loadings lower than 0.40, while item 16 was deleted due to ambiguity. Thirty-seven items remained following this procedure. After rerunning EFA (Table 4), eight factors emerged with eigenvalues of 1.00 (Nunnally, 1978), accounting for 67.03% of the variance, exceeding the minimum acceptable target of 60% for scale development (Hinkin, 1998). The examination of the factor structure revealed eight clearly distinct sets of items reflecting psychological feelings, sport/physical education policy, others’ expectations, appearance, affiliation, mastery, health benefits, and competition.

**Table 4:** Exploratory factor analysis (EFA) for the factors of the CUSPAM.

Eight-factor model				Nine-factor model			
Factor	Cronbach’s *α*	Item	Loading	Factor	Cronbach’s *α*	Item	Loading
Psychological condition	0.91	C-17	0.85	Stress management	0.85	C-17	0.81
		C-26	0.88			C-26	0.71
		C-08	0.74			C-08	0.85
		C-34	0.72			C-34	0.41
		C-32	0.72	Enjoyment	0.86	C-32	0.61
		C-05	0.62			C-05	0.54
		C-38	0.59			C-38	0.66
		C-23	0.45			C-23	0.90
Policy intervention	0.82	C-35	0.79	Policy intervention	0.82	C-35	0.79
		C-18	0.72			C-18	0.72
		C-09	0.64			C-09	0.64
		C-27	0.67			C-27	0.67
Others’ expectations	0.68	C-06	0.77	Others’ expectations	0.68	C-06	0.77
		C-15	0.55			C-15	0.55
Appearance	0.83	C-30	0.87	Appearance	0.83	C-30	0.87
		C-03	0.86			C-03	0.86
		C-21	0.75			C-21	0.75
		C-40	0.61			C-40	0.61
		C-12	0.40			C-12	0.40
Affiliation	0.84	C-13	0.90	Affiliation	0.84	C-13	0.90
		C-04	0.81			C-04	0.81
		C-31	0.54			C-31	0.54
Mastery	0.79	C-07	0.82	Mastery	0.79	C-07	0.82
		C-25	0.74			C-25	0.74
		C-11	0.68			C-11	0.68
		C-02	0.62			C-02	0.62
		C-33	0.43			C-33	0.43
Health benefits	0.81	C-20	0.75	Health benefits	0.81	C-20	0.75
		C-36	0.74			C-36	0.74
		C-19	0.59			C-19	0.59
		C-01	0.53			C-01	0.53
		C-28	0.43			C-28	0.43
		C-10	0.43			C-10	0.43
Competition	0.77	C-41	0.74	Competition	0.79	C-41	0.74
		C-37	0.66			C-37	0.66
		C-39	0.63			C-39	0.63
		C-29	0.59			C-29	0.59
	0.93				0.93		

*N* = 311. Extraction Method: Principal Axis Factoring. Rotation Method: Promax with Kaiser normalization. Only factor loadings greater than 0.40 are presented.

From the output of the EFA, two issues emerged to which we paid attention. First, all items clearly loaded on different factors. However, the originally designed two factors related to “stress management” and “enjoyment” converged into one factor, a result that was somewhat different from the expected factor structure. From a definition point of view, enjoyment is a positive emotion experienced by an individual after a satisfying activity (Abraham, 1943). While stress management implies a more specific process of cognitive appraisal to determine whether an individual believes he or she has the resources to respond effectively to the challenges of a stressor or a change (Folkman and Lazarus, 1988), it must be mentioned that both factors fall under the umbrella of psychological feelings. Therefore, we temporarily named this factor “psychological condition” in the eight-factor model in the subsequent analysis. However, this factor structure based on the EFA contradicted the traditional SDT classification of motivation types. In the early developmental stage of SDT, Deci (1975) proposed that people engage in an activity because they enjoy the activity and categorized this type of motivation as part of intrinsic motivation. Subsequently, this type of motivation is common among people from different social systems, cultures, and nationalities (Frederick and Ryan, 1993; Chen et al., 2006; Kueh et al., 2019). Stress management, on the other hand, is a type of extrinsic motivation related to the body (Rogers and Morris, 2003). Interestingly, items that originated from two different motivation types converged into one factor in the EFA. We speculate that the reason for this is that the EFA involved a preliminary exploration to identify the underlying relationships between measured variables without imposing any preconceived structure on the outcome (Child, 1990).

Therefore, it was necessary to conduct a follow-up detailed review of the eight overlapping items. These eight items were rerun in EFA with the extraction of two fixed factor numbers to investigate whether the loadings on each factor made any sense. The final EFA output showed that four items related to stress management and four items related to enjoyment loaded onto two factors separately, which is consistent with the proposed structure. Hence, two form models (eight-factor model and nine-factor model) were proposed and later tested using CFA to produce the final structural model.

Second, it should be noted that only two items representing others’ expectations were generally considered weak or unstable. However, Eisinga et al. (2013) explained that it is not uncommon for a questionnaire to have no more than two indicators to gage a particular self-assessment. This situation might be caused by two reasons. One is that due to resource and survey time constraints, only a limited number of items were available to assess a specific structure. The other is that poor-quality items were removed from the limited item pool, resulting in a small number of items in the scale, with only two items occasionally remaining. Given the high factor loading of these two items, i.e., item 6 (“Because my friends want me to”) and item 15 (“Because people tell me I need to”), we retained them for subsequent analysis.

### Confirmatory Factor Analysis

Summary statistics for the CFA of the tested eight-factor and nine-factor models are presented in Table 5. The factor “psychological condition,” composed of eight items in the eight-factor structure model, was split into two factors—“enjoyment” and “stress management”—in the nine-factor structure model. The factor loading of six items was increased (except item 32), which indicated that these items better reflected their respective latent variables and were highly correlated. By comparing the model matching index, it was obvious that all of the indices in the nine-factor model were higher than those in the eight-factor model, thereby demonstrating that the nine-factor model fit the data better. Considering the goodness of fit and the interpretability of solutions, the nine-factor model was the most appropriate for the current data.

**Table 5:** Comparison of the model fit indices.

Path model	*χ* ^2^	*χ*^2^/df	CFI	TLI	RMSEA	RMSEA (90% CI)
Eight-factor model	1655.60^***^	2.75	0.85	0.84	0.07	0.06–0.07
Initial nine-factor model	1506.91^***^	2.54	0.87	0.86	0.06	0.06–0.07
Modified nine-factor model	1009.58^***^	2.35	0.90	0.89	0.06	0.06–0.07
Final nine-factor model	946.06^***^	2.23	0.92	0.90	0.06	0.06–0.07

*** *p* < 0.001.

*N* = 330. CFI, comparative fit index; TLI, Tucker-Lewis index; RMSEA, root mean square error of approximation. 90% CI, lower boundary of a two-sided 90% confidence interval for the population and upper boundary of a two-sided 90% confidence interval for the population. Items 1, 2, 9, 12, and 19 were deleted due to the low factor loading in the modified nine-factor model. The final nine-factor model with correlated item residuals of the same domain: e1 and e2, e9 and e10, e24 and e26.

Although the values for the RMSEA and the *χ*^2^/df in the initial nine-factor model were acceptable, the values on the CFI and TLI were below the minimum acceptable levels. Therefore, based on recommendations by Hair et al. (2010), items with loadings below 0.70 were screened. After removing item 9 (“Because I want to meet the physical activity time required by the university”), item 12 (“Because I want to define my muscles so that I look better”), item 2 (“Because I like to engage in activities that physically challenge me”), item 19 (“Because I want to improve my cardiovascular fitness”), and item 1 (“Because I want to be physically fit”) due to low factor loading values that were considered insignificant, the CUSPAMS was reduced further, from 37 items to 32 items, while maintaining sufficient representation of the original variables with lower loadings. For the other six items (items 6, 15, 27, 29, 40, and 41), because their loadings were close to 0.70, and because, if deleted, their corresponding factors would only have two items, which would affect the subsequent internal consistency analysis (Hair et al., 2006), we decided to retain them. The modified CUSPAMS with 32 items revealed a data fit of *χ*^2^/df = 2.359, CFI = 0.91, TLI = 0.89, and RMSEA = 0.06, with adequate factor loadings as shown in the final nine-factor model. Further investigation improved the modified model by correlating the items’ residuals within the same latent variable. According to the modification indices, covariance for correlated items’ residuals was added to three pairs of items (items 8 and 17, items 18 and 27, and items 20 and 36) to produce the final version. The CFI and TLI values in the final model were all greater than 0.90, while the RMSEA was less than 0.08, which indicated an adequate fit for the expected nine-factor model for the Chinese sample (Table 5). Standardized factor loadings on all factors of the CUSPAMS ranged from 0.53 to 0.98, which were statistically significant (*p* < 0.001). This model also obtained better-fitting data in Sample 3 (*χ*^2^ = 1,723.84, df = 419, *p* < 0.001, CFI = 0.91, TLI = 0.90, RMSEA = 0.07).

**Figure 2:**
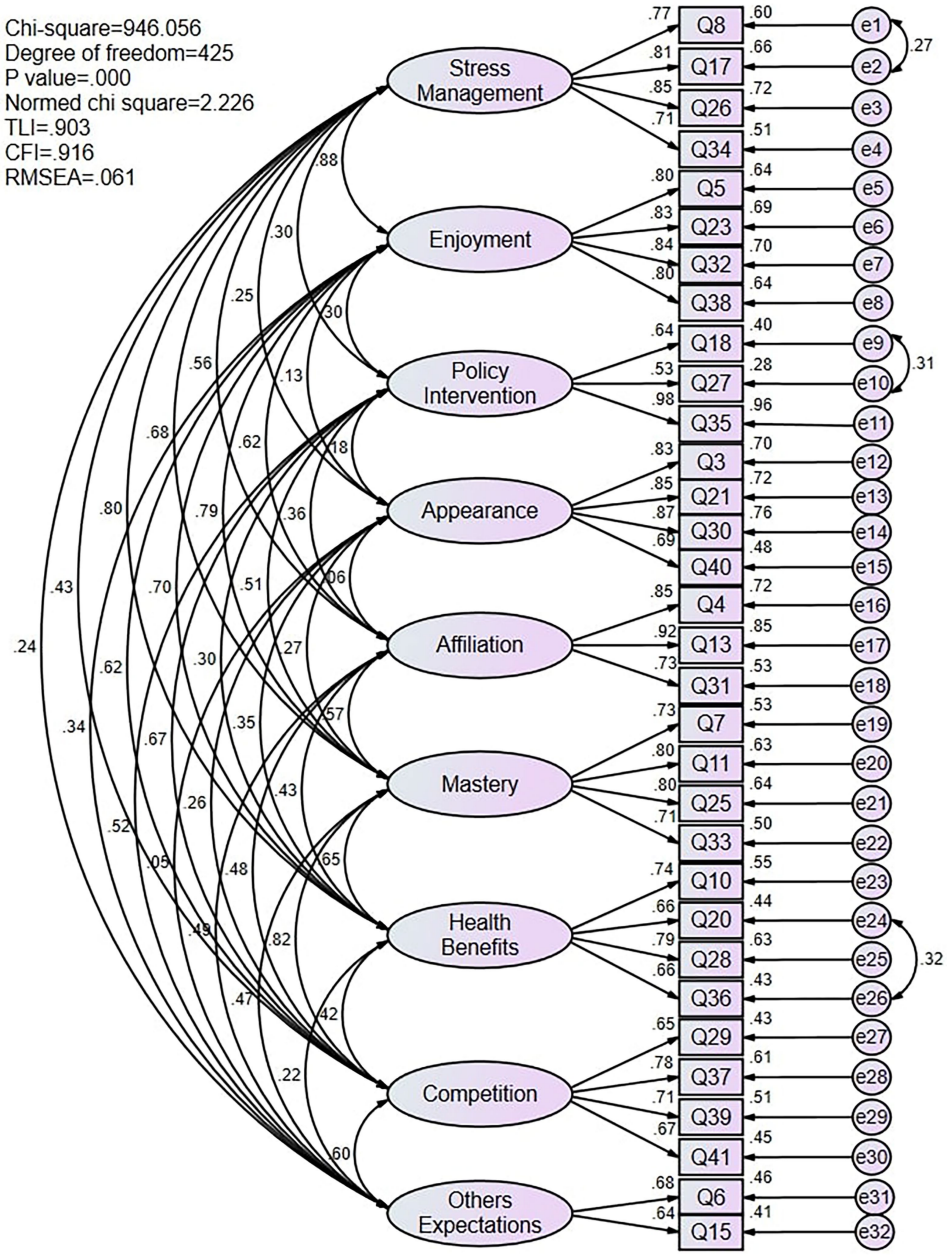
Measurement model for CUSPAMS.

The combined results of EFA and CFA led to the emergence of a model of nine motivational goals corresponding to the model framework proposed by the authors. But unfortunately, during the factor extraction process of the EFA, the two factors of enjoyment and stress management loaded onto one factor. We thus compared the model fitting indices of the two models through CFA and obtained the final nine-factor model version. Therefore, H1 was only partially confirmed (Figure 2).

### Reliability Analysis

The internal consistency of the CUSPAMS was assessed using Cronbach’s *α* and McDonald’s *ω*. The Cronbach’s *α* for the whole scale was 0.93 and, for the nine factors, varied from 0.79 to 0.88; meanwhile, McDonald’s *ω* ranged from 0.79 to 0.89. These results indicate high reliability of the nine factors of the CUSPAMS (Table 6). The internal consistency coefficient of the “others’ expectations” factor of only two items was not ideal, which was attributable to the sensitivity of Cronbach’s *α* to the number of items in the short scale. In this case, it may be more appropriate to report the mean inter-item correlation for the items (Pallant, 2010). After calculation, the inter-item correlation between item 6 and item 15 was 0.43, which is close to the optimal range of between 0.20 and 0.40 proposed by Briggs and Cheek (1986). The test–retest correlation coefficient for the total score of the CUSPAMS was 0.753, indicating that the CUSPAMS had higher stability.

**Table 6:** Composite reliability (CR), average variance extracted (AVE), Cronbach’s *α*, factor mean (SD), and factor correlation of final nine-factor model for the CUSPAMS.

	CR	AVE	*α*	*ω*	1	2	3	4	5	6	7	8	9
1. Enjoyment	0.88	0.66	0.88	0.89	4.26 (1.57)								
2. Stress management	0.87	0.63	0.86	0.87	0.76^**^	4.87 (1.38)							
3. Others’ expectations	0.60	0.43	0.60	-	0.24^**^	0.18^**^	2.33 (1.24)						
4. Appearance	0.88	0.59	0.88	0.79	0.11^*^	0.21^**^	0.04	5.18 (1.57)					
5. Affiliation	0.87	0.70	0.86	0.87	0.57^**^	0.50^**^	0.37^**^	0.07	3.71 (1.61)				
6. Mastery	0.84	0.57	0.84	0.85	0.69^**^	0.57^**^	0.33^**^	0.21^**^	0.53^**^	3.88 (1.53)			
7. Health benefits	0.82	0.54	0.82	0.82	0.50^**^	0.57^**^	0.14^**^	0.23^**^	0.32^**^	0.48^**^	5.34 (1.22)		
8. Competition	0.79	0.49	0.79	0.80	0.51^**^	0.36^**^	0.42^**^	0.24^**^	0.41^**^	0.63^**^	0.35^**^	2.72 (1.31)	
9. Policy intervention	0.78	0.55	0.79	0.79	0.17^**^	0.21^**^	0.38^**^	0.17^**^	0.33^**^	0.36^**^	0.21^**^	0.47^**^	4.17 (1.62)

* Correlation is significant at the 0.05 level (two-tailed). McDonald’s *ω* = 0.93. Cronbach’s *α* = 0.93.

** Correlation is significant at the 0.01 level (two-tailed).

### Convergent and Discriminant Validity

The convergent validity of the measurement model was established by calculating the average variance extracted (AVE) and composite reliability (CR). These indicators can reflect the degree of shared variance between latent variables. Fornell and Larcker (1981) suggested that if the value of the AVE was greater than 0.70, it should be considered very good, whereas a level of 0.50 is acceptable; meanwhile, a CR value greater than or equal to 0.70 is considered acceptable. Based on the final nine-factor model, in addition to the others’ expectations factor, the CR values of the other eight factors ranged from 0.78 to 0.88, and the AVE values ranged from 0.49 to 0.70. Hence, we concluded that the convergent validity of the construct was adequate. The values of CR, AVE, and correlation coefficients are shown in Table 6. The factor correlations that were not significant were others’ expectations with appearance, and affiliation with appearance. However, other pairs were significant.

Recently, the heterotrait-monotrait ratio of correlations (HTMT) approach was proposed to assess discriminant validity (Henseler et al., 2015). HTMT is derived from the classic multitrait-multimethod (MTMM) matrix (Campbell and Fiske, 1959), which is an estimate of the correlation between constructs. Henseler et al. (2015) proposed that HTMT values lower than 0.90 show that the true correlation between the two constructs should differ. From the calculation results (Table 7), all correlations achieved the recommended value of below 0.90. This indicated that the final nine-factor model of the CUSPAMS showed good discriminant validity.

**Table 7:** Heterotrait-monotrait ratio (HTMT) of nine-factor model in CUSPAMS analysis.

	1	2	3	4	5	6	7	8	9
1. Stress management									
2. Enjoyment	0.87								
3. Policy intervention	0.27	0.20							
4. Others’ expectations	0.24	0.33	0.49						
5. Appearance	0.24	0.13	0.23	0.06					
6. Affiliation	0.57	0.65	0.37	0.51	0.82				
7. Mastery	0.68	0.79	0.45	0.48	0.29	0.61			
8. Health benefits	0.76	0.67	0.23	0.23	0.31	0.44	0.63		
9. Competition	0.42	0.60	0.61	0.62	0.29	0.49	0.81	0.41	

### Criterion-Related Validity

The correlation matrix between the CUSPAMS and C-BREQ-2 subscales showed that enjoyment, mastery, health benefits, stress management, appearance, competition and intrinsic motivation, identified regulation were highly positively correlated, while policies, others’ expectations and amotivation, external regulation were highly positively correlated (see Table 8). Slightly different from the expected hypothesis, H2, appearance was related to a more autonomous form of regulation. In addition, the CUSPAMS also correlated with the SEE-C. Except for policy intervention, others’ expectations and appearance were weakly correlated with the SEE-C, and the remaining six factors were significantly and moderately correlated with the SEE-C. These results confirm hypotheses H2 and H3.

**Table 8:** Pearson’s correlation coefficients between the CUSPAMS subscales and different dimensions of C-BREQ-2 and SEE-C (sample 3).

Factors	Amotivation	External regulation	Introjected regulation	Identified regulation	Intrinsic motivation	Self-efficacy
Stress management	0.021	0.154^**^	0.388^**^	0.586^**^	0.780^**^	0.560^**^
Enjoyment	0.08	0.186^**^	0.474^**^	0.606^**^	0.830^**^	0.579^**^
Policy intervention	0.204^**^	0.362^**^	0.376^**^	0.315^**^	0.277^**^	0.270^**^
Appearance	0.117^**^	0.275^**^	0.376^**^	0.439^**^	0.436^**^	0.388^**^
Affiliation	0.237^**^	0.367^**^	0.425^**^	0.414^**^	0.587^**^	0.455^**^
Mastery	0.092^*^ [Table-fn tfn7]	0.228^**^	0.527^**^	0.615^**^	0.701^**^	0.573^**^
Health benefits	−0.039	0.162^**^	0.451^**^	0.715^**^	0.688^**^	0.508^**^
Competition	0.340^**^	0.461^**^	0.527^**^	0.414^**^	0.529^**^	0.512^**^
Others’ expectations	0.383^**^	0.494^**^	0.315^**^	0.099^*^	0.175^**^	0.290^**^

* *p* < 0.05;

** *p* < 0.01.

## Discussion

Chinese university students who are under strict school PE policy may show more “complicated” PA motivations than university students in other countries. The current literature lacks an instrument that can specifically measure this unique motivation among Chinese university students to engage in PA, which represents a unique methodological extension to understand motivation research. Initially, we developed an item pool of 41 motives based on the theoretical framework of SDT, after which we explored and validated the structure of these items with a factor analytic approach. The EFA results yielded eight meaningful factors, but the two factors of enjoyment and stress management loaded on the same factor. However, the degree of autonomy of these two motives in SDT theory is not the same. Therefore, in order to resolve the conflict between this statistical result and SDT theory, we relied on CFA to compare the model fit of the two models with Sample 2. Compared with the eight-factor model, the factor loading and model fit index of the nine-factor model (e.g., CFI, TLI, and RMSEA) were all improved, showing better structural validity. However, the reasons for the high correlation between enjoyment and stress management factors must be analyzed further in future research.

The reliability of the CUSPAMS was supported by the indices obtained for internal consistency and temporal stability. All of the Cronbach’s *α* and McDonald’s *ω* of the factors were above 0.70 except Others’ Expectations. It is worth noting that the items of Others’ Expectations in this study were extracted from the PALMS. In the cross-cultural adaption studies of the PALMS, the authors made different choices on whether to delete or retain this factor. The design of these items is more suitable for a population sample with a large age range, but it does not show satisfactory reliability when only applied to student groups (Hu et al., 2019; Santos-Labrador et al., 2021). In this study, due to the high factor loading, we chose to retain Q6 and Q15 in the final version to expand the range of motivation types. In future practical research of the CUSPAMS, it will be necessary to observe the statistical performance of this factor to decide how to take subsequent action. In addition, the PA motivation measured by the CUSPAMS was stable for a fairly long period of 3 weeks. Regarding the convergent validity of the CUSPAMS, except for Others’ Expectations, the CR and AVE values of the remaining eight factors all met the recommended criteria, indicating that the loadings of each factor were well accounted for, and that each factor was internally consistent.

Evidence of criterion validity was provided by correlations between each factor score of the CUSPAMS and scores on the factor of C-BREQ-2 and SEE-C, with only one exception: Appearance was associated with more self-determined regulation. In fact, the introjected regulation of avoiding guilt and shame may be particularly important and is generally regarded as a potential positive motivation for exercise behavior change. Two previous studies have revealed evidence that the appearance motive is highly correlated with introverted accommodation, especially for women who are more prone to guilt (Castonguay et al., 2015; Hurst et al., 2017). However, the exercise motives of individuals seeking to improve their appearance may show an overlap of controllable motivation and non-controllable motivation. For example, a man may aim to achieve a physically appealing body because his partner compliments his good looks (controlled motivation) and, at the same time, he may personally value a fit appearance (autonomous motivation; Ingledew and Markland, 2008). As a result, the positive relationship between appearance motives and PA participation may have been generated by the autonomous framing of appearance-related goals.

Many sports enthusiasts or casual exercisers are undoubtedly triggered by both intrinsic and extrinsic motivations, but may differ in the relative salience of these different foci (Ryan et al., 1997). From the output of the EFA results, it can be seen that the motivations of university students to participate in PA are diverse, and the intensity distribution on the nine dimensions of the CUSPAMS are different. In addition to policy interventions, the motivational goals mentioned in SDT and other motivational scales can be found in Chinese samples, which demonstrates that motivation types under different cultural backgrounds are based on stable factor invariance. The newly added factor, i.e., policy intervention or sport education policy in the CUSPAMS, which is relevant to the context of China’s unique education PE policies and regulations, is a valuable inclusion to understand theory related to extrinsic motivation.

From the mean score of the factors in Table 6, the mean of the policy intervention factor was slightly behind the enjoyment factor and ahead of the mastery factor. Both enjoyment and mastery are intrinsic motivations of an autonomous form, which have been demonstrated to be highly related to PA adherence (Mullan and Markland, 1997). This shows that many Chinese university students are more responsive to items related to policy intervention. In other words, the motivation of these students to participate in PA is to avoid the potential punishments imposed by PE policies. Ryan and Deci (2000) proposed that the stability of one’s motivation is at least partially dependent on some of its qualitative features, particularly the degree of perceived autonomy or an internal perceived locus of causality. According to SDT theory, motivation driven by PE policy is clearly a typical controlled form of extrinsic motivation. Although it sometimes regulates (or motivates) short-term exercise behavior, it cannot be sustained over time (Deci and Ryan, 1985; Ryan et al., 1997). A recent study in China reported that university students’ attitudes toward school PE policies are positively correlated with their scores on certain physical tests. The ignorance and disapproval of policies have caused a small number of Chinese university students to fear or even avoid participating in related PA activities (Chen et al., 2016). If university students participate in PA to avoid policy punishment rather than because they want to, then this will eventually lead to a higher dropout rate, and this form of motivation is not conducive to the formation of healthy exercise habits (Wang, 2001). Therefore, the accurate capture of policy-related motivations may be the key to promoting policy adjustments and changing student PA behavior. Policymakers and health management departments need to look more closely at goals and self-regulatory features associated with regular participation in exercise and PA. The development of the CUSPAMS provides a valid scale to identify motivations in university students, one that could potentially assist policymakers in understanding PA motivation patterns, thus improving the effectiveness of PE policy intervention and developing lifelong exercise habits that will persist beyond university.

Some limitations should also be mentioned. First, the data were collected based on self-reports, which means that the participants may have been influenced by “social desirability,” consciously or unconsciously. In addition, the time spent completing the questionnaire may have led to fatigue or boredom. These are potential factors that affect the quality of the data. However, the data provided in this study demonstrated acceptable reliability and validity. In future research on the CUSPAMS, efforts should be made to develop more effective strategies to obtain comprehensive data from university students, such as employing mixed methods, e.g., interviews. Second, the statistical techniques of EFA and CFA used in this study only provided the construct validity of the CUSPAMS with nine factors. However, after these structures have been identified, future research must use item response theory (IRT) to investigate the quality of each item in each factor. Because IRT is based on a survey of whether the tool meets a set of assumptions, it provides more detailed information about the effectiveness of the measurement tool (Hays et al., 2000; Ye et al., 2018b, 2019). This is an effective supplement to the psychometric characteristics of the initial CUSPAMS. Third, in general, the implementation target population of Chinese PE policy is toward all students, ranging from primary school to university. However, this study only focused on the PA motivation of university students, and hence the outcome of using the CUSPAMS on non-university students must be assessed in future studies. Considering the huge population of Chinese university students, the application of the CUSPAMS is still of great practical significance for understanding the motivation types of university students. Finally, the CUSPAMS is not applicable in countries that do not require mandatory PE classes in university settings or scores on physical fitness tests as part of graduation criteria. Hence, this final limitation could be viewed as a limitation yet could be viewed positively as the uniqueness or novelty of CUSPAMS in terms of understanding policy-driven motivation on PA.

## Conclusion

The final measurement model of the nine-factor CUSPAMS comprised 32 items. Evidence for content validity, construct validity, face validity, and internal consistency reliability were presented. The results of this study revealed that the CUSPAMS provides adequate evidence of validity and reliability. This scale sufficiently captured a wider range of PA motivation in a population of Chinese university students. Also, the factors of policy intervention or government PE policies in the CUSPAMS encompassed a wider extension of extrinsic motivation in SDT theory. This study provides new insights into the influence of government policies on PA motivation. Future research on motives for participation can use the CUSPAMS to examine the motives for engaging in any form of PA, interpreting responses within the nine-factor framework.

## Data Availability Statement

The raw data supporting the conclusions of this article will be made available by the authors, without undue reservation.

## Ethics Statement

The studies involving human participants were reviewed and approved by Ethics Committee of the University of Malaya (UM. TNC2/UMREC-976). The patients/participants provided their written informed consent to participate in this study.

## Author Contributions

ET contributed to the conception and made a major contribution to the manuscript revision process. BL performed the statistical analyses and drafted the manuscript. TY was in charge of data collection and assisted in statistical analyses. All authors read and approved the final manuscript.

## Conflict of Interest

The authors declare that the research was conducted in the absence of any commercial or financial relationships that could be construed as a potential conflict of interest.

## Publisher’s Note

All claims expressed in this article are solely those of the authors and do not necessarily represent those of their affiliated organizations, or those of the publisher, the editors and the reviewers. Any product that may be evaluated in this article, or claim that may be made by its manufacturer, is not guaranteed or endorsed by the publisher.

## Abbreviations:

PA: Physical activity
PE: Physical education
CUSPAMS: Chinese university students’ physical activity motivation scale
SDT: Self-determination theory
EFA: Exploratory factor analysis
CFA: Confirmatory factor analysis.
